# Low saliva pH can yield false positives results in simple RT-LAMP-based SARS-CoV-2 diagnostic tests

**DOI:** 10.1371/journal.pone.0250202

**Published:** 2021-05-05

**Authors:** Cristina Uribe-Alvarez, Quynh Lam, Don A. Baldwin, Jonathan Chernoff

**Affiliations:** 1 Cancer Signaling and Epigenetics Department, Fox Chase Cancer Center, Philadelphia, Pennsylvania, United States of America; 2 Department of Pathology, Fox Chase Cancer Center, Philadelphia, Pennsylvania, United States of America; University of Helsinki, FINLAND

## Abstract

Diagnosis of any infectious disease is vital for opportune treatment and to prevent dissemination. RT-qPCR tests for detection of SARS-CoV-2, the causative agent for COVID-19, are ideal in a hospital environment. However, mass testing requires cheaper and simpler tests, especially in settings that lack sophisticated machinery. The most common current diagnostic method is based on nasopharyngeal sample collection, RNA extraction, and RT-qPCR for amplification and detection of viral nucleic acids. Here, we show that samples obtained from nasopharyngeal swabs in VTM and in saliva can be used with or without RNA purification in an isothermal loop-mediated amplification (LAMP)-based assay, with 60–93% sensitivity for SARS-CoV-2 detection as compared to standard RT-qPCR tests. A series of simple modifications to standard RT-LAMP published methods to stabilize pH fluctuations due to salivary acidity resulted in a significant improvement in reliability, opening new avenues for efficient, low-cost testing of COVID-19 infection.

## Introduction

The years 2019 to 2021 will be remembered for the coronavirus-disease 2019 (COVID-19) pandemic. The disease toll in the world has surpassed 142.5 million cases and more than 3 million deaths and does not seem to have decreased its rate of contagion in the past months [1]. In the US alone more than 31.7 million cases and over five hundred and sixty eighty thousand deaths have been reported [1]. Case fatality ratio in the US is around 1.8% (number of deaths/number of confirmed cases), in developing countries such as Brazil, the fatality ratio is around 2.7%, while in Mexico it goes as high as 9.2%. Simple, inexpensive, and accurate diagnostic techniques are of utmost importance to isolate infected individuals and slow down the transmission of the disease, prevent oversaturation of health care facilities and attenuate morbidity and mortality. Accordingly, the scientific community has made remarkably quick strides to develop diagnostic tools, either for use in specialized health centers or for point-of-care community outposts [2].

Testing for SARS-CoV-2, the causal agent of COVID-19, is usually based on detecting proteins (viral antigens or host antibodies) or viral nucleic acids. Antibody detection tests indicate if the person has been infected by SARS-CoV-2 and has generated IgG and/or IgM antibodies. These tests are performed in blood serum or plasma and, while inexpensive and easy to administer, do not indicate if the infection is active [3, 4], as it can take from 1 to 3 weeks after exposure to produce enough antibodies to be detected [5, 6]. Antibody titers vary in patients; those that present milder symptoms or who are asymptomatic usually have relatively low antibody titers that disappear a few weeks after infection, while patients with more severe symptoms generally present higher antibody titers that may be detected two or three months after infection [7]. When performed in the correct stage of infection antibody test sensitivity may be around 90% and results may be obtained in as little as 15 minutes [3, 8].

Viral load-based tests detect viruses present in the host and can be either antigen-based, detecting specific fragments of viral proteins, or PCR-based, amplifying viral RNA. Unlike serological tests, these tests indicate if the patient has an active infection regardless of their immune response. Immunochromatographic antigen tests can yield results in 15 minutes. However, reported results range from 100% (based on 7 samples) to 32% accuracy (based on 106 positive RT-qPCR samples) [4, 8–10]. The current FDA-recommended method to determine COVID-19 infections is based on reverse transcription quantitative polymerase chain reaction (RT-qPCR). This approach to virus detection amplifies specific sequences from viral SARS-CoV-2 RNA found in a given sample. Depending on the manufacturer, the nature and volume of the sample, and the oligonucleotides, RT-qPCR tests can detect as few as 242 SARS-CoV-2 RNA copies/mL [11] or from 1 to 10 genomic copy equivalents per reaction [12].

There are three issues regarding standard RT-qPCR that make it less than ideal for large scale testing. First, the tests are usually performed using nasopharyngeal (NP) samples suspended in virus transport medium (VTM). As the sampling method is unpleasant, requires specialized swabs, and is difficult to self-administer, saliva sampling has been considered as an alternative source of specimens [13–15]. Second, extraction of RNA from the samples is tedious and adds considerable time and expense to the assay. And third, RT-qPCR tests generally require expensive kits and access to an expensive thermocycler that may not be available in all settings. We sought to address all three of these issues to develop a faster, less expensive, and more accessible testing platform for detection of SARS-CoV-2 RNA from patients.

## Methodology

### Patient samples

Residual samples were retained in a de-identified fashion with no link to patient identifiers. These remnant diagnostic swab samples from Fox Chase Cancer Center, Jeanes Hospital, and Temple University Hospital patients were stored in VTM at -80 °C after testing in the Fox Chase Molecular Diagnostics Laboratory. Saliva samples were obtained from healthy, consenting adult volunteers and stored at -80°C after pH measurments and RT-LAMP testing.

The SARS-CoV-2 diagnostic test used in the Fox Chase Molecular Diagnostics Laboratory extracts RNA from patient nasopharyngeal samples in VTM using a Qiagen QIAamp Viral or a Perkin Elmer chemagen Viral 300 kit, followed by RT-qPCR in an ABI QuantStudio 12K Flex instrument using the ThermoFisher TaqPath COVID-19 Combo Kit, that can detect at least 10 copies of virus per reaction. SARS-CoV-2 is stable and can be detectable by RT-qPCR and LAMP in both VTM and saliva for 7 to 25 days at a range of 4 to 30°C [13, 16].

### Protocols for nasopharyngeal (NP) and saliva samples

#### Direct assay

100X of inactivation buffer (0.5 M of TCEP-HCl, 0.1 M EDTA< pH 8, plus 1.15 N of NaOH, 0.1% μL of NP-10 and 5% of SDS in MQ water, pH 8 with NaOH) was added to treat the samples using the direct assay. Limit detection curves were made using diferent dilutions from the TaqPath COVID-19 RNA control, A47814 ThermoFisher Scientific. Samples were immediately vortexed, pulse-centrifuged, incubated at 95°C for 5 minutes and centrifuged 30s at 5,000 xg to precipitate the proteins in the VTM and saliva samples. The addition of detergents and heating ensures killing the virus. 1.0 μL of this supernatant was added to a previously set up 10 μL RT-LAMP reaction.

#### RNA precipitation assay

Nucleotides present in the sample were precipitated using silica beads [17]. Briefly, NP samples in VTM or saliva were added to an Eppendorf tube containing a solution with 100X inactivation buffer, and RNAsecure (25X) [18]. The addition of the RNAsecure (Beta-mercaptoethanol mix), irreversibly denatures RNAses by reducing disulfide bonds therefore protecting RNA. For saliva samples, one microliter of proteinase K (MEB 8107S) 1:10 dilution was added per 250 μL reaction [18]. Samples were vortexed, pulse-centrifuged, incubated at 55°for 15 min and 95°C for 5 minutes, and centrifuged 30s at 5,000 xg to precipitate the unwanted protein. Treated samples were transferred to a new tube, taking care to avoid carry over of the precipitate. We added 0.35 mL of RNA binding solution (6M of NaI, 2% Triton-100 and 10 mM HCl) and 5 μL of glass milk/silica gel 1:1 w/v in 10 mM Tris-HCl pH 8 and 1 mM EDTA pH 8, per 0.75 mL of sample and left at room temperature during 15–20 minutes shaking carefully by inversion every two minutes. Samples were centrifuged 1 min at maximum speed in a microcentrifuge. The supernatant was discarded in 10% bleach and the pellet was washed with 80% EtOH without dislodging it. Samples were centrifuged for 1 min at 13,000 xg, then ethanol was discarded and tubes were dried at 55°C for one minute. Samples were resuspended in 9 μL of preheated 1x inactivation buffer and used for the RT-LAMP assay or kept at -80°C. 3 μL of this sample were added directly to a previously set up 10 μL RT-LAMP reaction.

### RT-LAMP reaction

Reactions were set up according to the WarmStart LAMP Kit (NEB). First the LAMP master mix was added to the PCR tubes to avoid contamination. We used two or three sets of oligos for each assay: NEB Gene N-A, HMS Assay 1e, NEB orf1a-A oligonucleotides, and an actin control (ACTB) for saliva samples. Primers were designed for specific genes from the genome of the SARS-CoV-2.

LAMP primers.

$$\begin{array}{ll} \text{NEB}\;\text{orf}1\text{a}-\text{A}-\text{F}3 & \text{CTGCACCTCATGGTCATGTT}\\ \text{NEB}\;\text{orf}1\text{a}-\text{A}-\text{B}3 & \text{AGCTCGTCGCCTAAGTCAA}\\ \text{NEB}\;\text{orf}1\text{a}-\text{A}-\text{FIP} & \text{GAGGGACAAGGACACCAAGTGTATGGTTGAGCTGGTAGCAGA}\\ \text{NEB}\;\text{orf}1\text{a}-\text{A}-\text{BIP} & \text{CCAGTGGCTTACCGCAAGGTTTTAGATCGGCGCCGTAAC}\\ \text{NEB}\;\text{orf}1\text{a}-\text{A}-\text{LF} & \text{CCGTACTGAATGCCTTCGAGT}\\ \text{NEB}\;\text{orf}1\text{a}-\text{A}-\text{LB} & \text{TTCGTAAGAACGGTAATAAAGGAGC} \end{array}$$

$$\begin{array}{cc} \text{As}1\_\text{F}3 & \text{CGGTGGACAAATTGTCAC}\\ \text{As}1\_\text{B}3 & \text{CTTCTCTGGATTTAACACACTT}\\ \text{As}1\_\text{FIP} & \text{TTACAAGCTTAAAGAATGTCTGAACACT}\\ \text{As}1\_\text{BIP} & \text{TTGAATTTAGGTGAAACATTTGTCACG}\\ \text{As}1\text{e}\_\text{LF} & \text{TCAGCACACAAAGCCAAAAATTTAT}\text{TTTT}\text{CTGTGCAAAGGAAATTAAGGAG}\\ \text{As}1\text{e}\_\text{LB} & \text{TATTGGTGGAGCTAAACTTAAAGCC}\text{TTTT}\text{CTGTACAATCCCTTTGAGTG} \end{array}$$

$$\begin{array}{cc} \text{NEB}\;\text{Gene}\;\text{N}-\text{A}-\text{F}3 & \text{TGGCTACTACCGAAGAGCT}\\ \text{NEB}\;\text{Gene}\;\text{N}-\text{A}-\text{B}3 & \text{TGCAGCATTGTTAGCAGGAT}\\ \text{NEB}\;\text{Gene}\;\text{N}-\text{A}-\text{FIP} & \text{TCTGGCCCAGTTCCTAGGTAGTCCAGACGAATTCGTGGTGG}\\ \text{NEB}\;\text{Gene}\;\text{N}-\text{A}-\text{BIP} & \text{AGACGGCATCATATGGGTTGCACGGGTGCCAATGTGATCT}\\ \text{NEB}\;\text{Gene}\;\text{N}-\text{A}-\text{LF} & \text{GGACTGAGATCTTTCATTTTACCGT}\\ \text{NEB}\;\text{Gene}\;\text{N}-\text{A}-\text{LB} & \text{ACTGAGGGAGCCTTGAATACA} \end{array}$$

$$\begin{array}{cc} \text{ACTB}-\text{F}3 & \text{AGTACCCCATCGAGCACGACT}\\ \text{ACTB}-\text{B}3 & \text{AGCCTGGATAGCAACGTACAACT}\\ \text{ACTB}-\text{FIP} & \text{GAGCCACACGCAGCTCATTGTATCACCAACTGGGACGACAACT}\\ \text{ACTB}-\text{BIP} & \text{CTGAACCCCAAGGCCAACCGGCTGGGGTGTTGAAGGTCACT}\\ \text{ACTB}-\text{LF} & \text{TGTGGTGCCAGATTTTCTCCAACT}\\ \text{ACTB}-\text{LB} & \text{CGAGAAGATGACCCAGATCATGT} \end{array}$$

Primers master mix was prepared as described in [19]: 32 μM of each inner primer (FIP/BIP), 4 μM of each outer primer (F3/B3), 8 μM of each loop primer (LF/LB) were mixed in a 100 μL final volume. Primer sequence was obtained from [20] for NEB Gene N-A and NEB orf1a-A oligonucleotides, from [17] for HMS Assay 1e and from [21] for ACTB oligonucleotides.

The reaction mixture was 1 μL of oligonucleotide mix, 5 μL of WarmStart^®^ Colorimetric Master Mix, 3 or 1 μL of RNA template and nuclease free water to reach a final volume of 10 μL. After the reaction mixture was prepared, tubes were vortexed and centrifuged. Reaction mixtures were color pink or red. The presence of carried over silica beads in the sample did not affect the pH or the final SARS-CoV-2 result. Samples were incubated for 30 min at 65°C in PCR tubes in a Bio-Rad thermocycler. Absorbance was measured in a nanodrop device at 448 and 570 nm.

### Statistical analysis

Paired t-tests were used for comparison between the 448/570 absortion ratio of positive and negative samples used to determine the critical threshold value; and between the absorbance ratios registered at each concentration of SARS-CoV2 compared to a sample with no SARS-CoV2 in order to establish the RT-LAMP limit of detection. All hypothesis tests were two-sided with a 5% type I error. Sensitivity, specificity, positive predictive value and negative predictive value with two-sided exact 95% confidence intervals were computed to assess the operating characterstics of the direct assay and the RNA precipitation assay compared to the standard clinical (*i*.*e*. with RNA purification) RT-qPCR test. Statistical analyses were completed using GraphPad Prism 6.0 or 7.0 (La Jolla, CA, USA).

## Results

### Limit of detection for RT-LAMP based methods to detect SARS-CoV-2 viral RNA

The “gold-standard” test for detecting SARS-CoV-2 infection uses nasopharyngeal (NP) samples collected in virus transpot medium (VTM) followed by RNA extraction and viral gene amplification and detection by RT-qPCR [22, 23]. We sought to simplify all three parts of this procedure.

**Fig 1:**
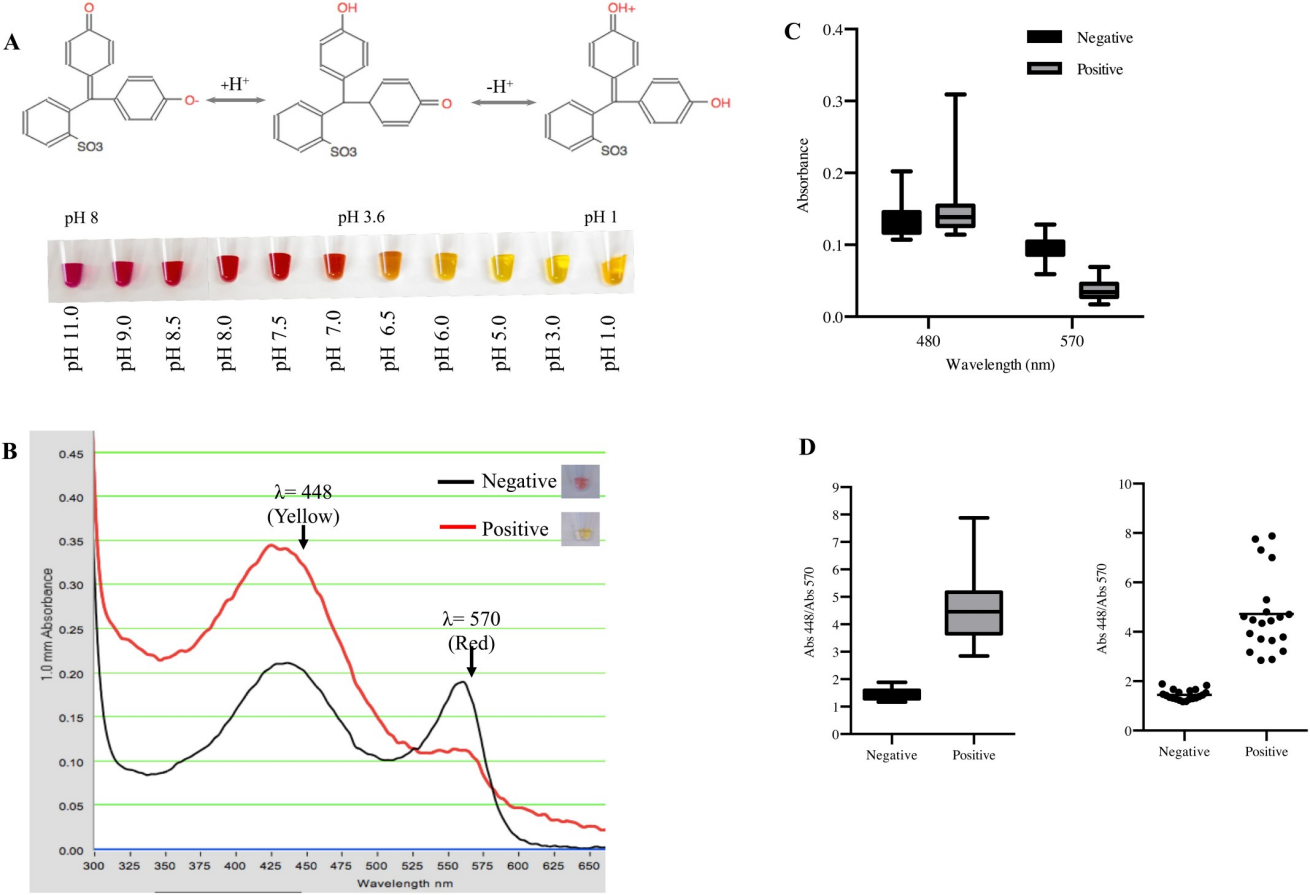
Critical value threshold determination for RT-LAMP tests for SARS-CoV-2 detection. A) NEB WarmStart LAMP kit pH is monitored using the pH indicator phenol red. In acid media phenol red has a yellow color and as the pH rises it turns to orange, red and finally pink. Addition of a new complementary nucleotide (dNTP) to a new synthesized DNA chain will form a phosphodiester bond between the α phosphate of the 3’ hydroxide of the pentose acidifying the medium and therefore turning the reaction color from red (basic) to yellow (acidic). B) Representative absorption spectrum from a negative and positive SARS-CoV-2 spiked sample using the NEB kit LAMP. The absorption spectrum for the negative sample is shown in a black line and the positive sample is shown in red line. Measurements were taken at two absorption maximum points, one in yellow (λ = 448 nm) and one in red (570 nm). C) Box plots represent the absorbance values of positive viral RNA-spiked samples and negative samples at 448 and 570 nm (n = 20) D) The quotient of 448/570 nm of negative and positive samples was used to set the crisitcal value threshold at 2. Box plots represent the values between positive and negative SARS-CoV2 spiked samples. Paired t-test of n = 20 **** P <0.0001.

Warm Start colorimetric RT-LAMP (New England Biolabs, M1800L) assays can detect viral RNA using a single temperature, can be completed in half an hour, and can provide a colorimetric readout, obviating the need for a thermocycler or a device capable of real-time fluorescence measurements. We began by determining if an RT-LAMP-based approach might be a suitable substitute for RT-qPCR. To compare the limit of detection of both tests, we spiked known quantities of SARS-CoV-2 viral RNA (Dilutions from the TaqPath COVID-19 RNA control, A47814 ThermoFisher Scientific) directly into a previously setup RT-LAMP reaction with NEB Gene N-A oligonucleotides [19]. We then monitored the reaction over a 30-minute period for changes in the medium acidity which would tranlsate in a medium color shift from pink to yellow (Fig 1A). We measured the maximal absorbance of phenol red at 448 nm (yellow) in acidic conditions and at 570 nm (red) in basic conditions [24] (Fig 1B). We quantified the absorbance of the samples at these wavelengths and used the ratio between 448/570 to set the critical value that was used as a threshold to determine if a sample was positive or negative. Initial samples were pink/red in color; samples that lacked SARS-CoV-2 RNA, had no medium acidification and the 448/570 ratio remained below 2 at all times. Spiked positive samples amplified SARS-CoV-2 RNA switching colors from red to yellow and increasing the value of the 448/570 ratio. Samples with a ratio above our threshold value 2 were considered positive (Fig 1C and 1D).

**Fig 2:**
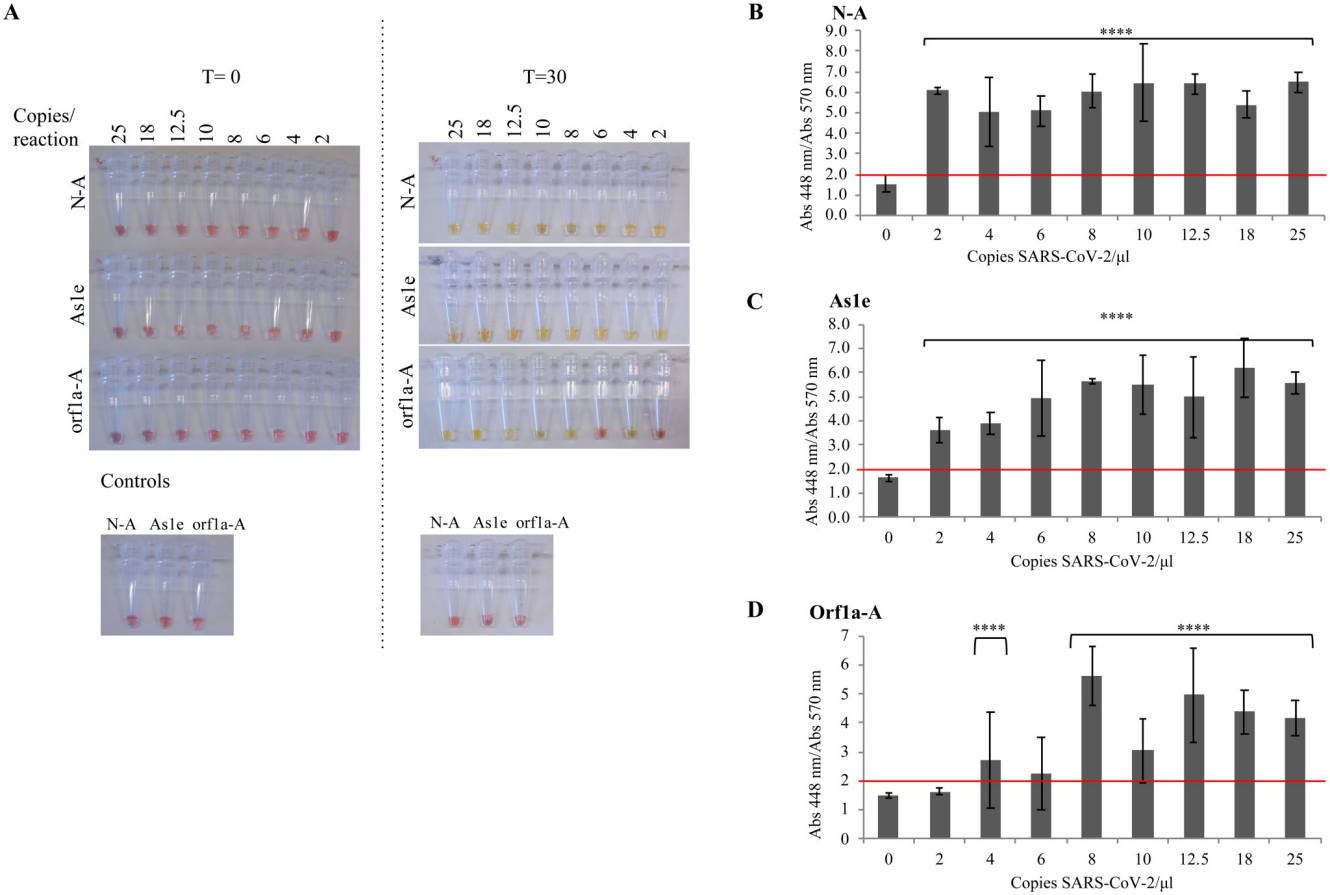
RT-LAMP assay limit of detection. Known concentrations of SARS-CoV-2 virus diluted in inactivation buffer were used to spike previously set up RT-LAMP reactions with oligonucleotide pairs NEB Gene N-A (N-A), HMS Assay 1e (As1e) and NEB orf1a-A (orf1a-A). Control samples with no virus were also monitored. LAMP tests were incubated at 65°C for 30 minutes in a Bio-Rad thermocycler and the resulting reaction was (A) imaged and the absorbance at 448 and 570 nm was measured. The absorbance quotient between 448/570 nm was used to distinguish positive versus negative samples for (B) oligonucleotide NEB Gene N-A, (C) oligonucleotide HMS Assay 1e and (D) oligonucleotide NEB orf1a-A. Statistics: Paired t-test between the absorbance ratio registered at each concentration of SARS-CoV-2 compared to a sample with no SARS-CoV-2. Values represent the mean ± S.D. of n = 3 for each concentration **** P <0.0001.

Having established the basic assay and critical value, we next used three different primer sets–NEB Gene N-A [20], NEB orf1a-A [18] and HMS Assay 1e (As1e) [17]–to evaluate if they could detect SARS-CoV-2 sequences. As in the previous assay, we added a known concentration of viral RNA directly into the RT-LAMP reaction tube and recorded the absorbance spectrum of the sample after a 30-minute incubation at 65°C (Fig 2, S1 Fig). NEB Gene N-A and HMS Assay 1e primers detected as few as 2 copies of viral RNA per reaction (Fig 2A–2C), while the NEB orf1a-A primers were less efficient, with a detection limit of 12.5 copies per reaction (Fig 2A and 2D).

### Comparison of direct *versus* RNA-purified methods for detection of spiked SARS-CoV-2 in VTM

SARS-CoV-2 viral RNA purification adds time, complexity, and increases the cost of the assay. We next sought to determine if the RT-LAMP reaction to detect artifitially spiked SARS-CoV-2 samples was imporved when RNA was extracted.

**Fig 3:**
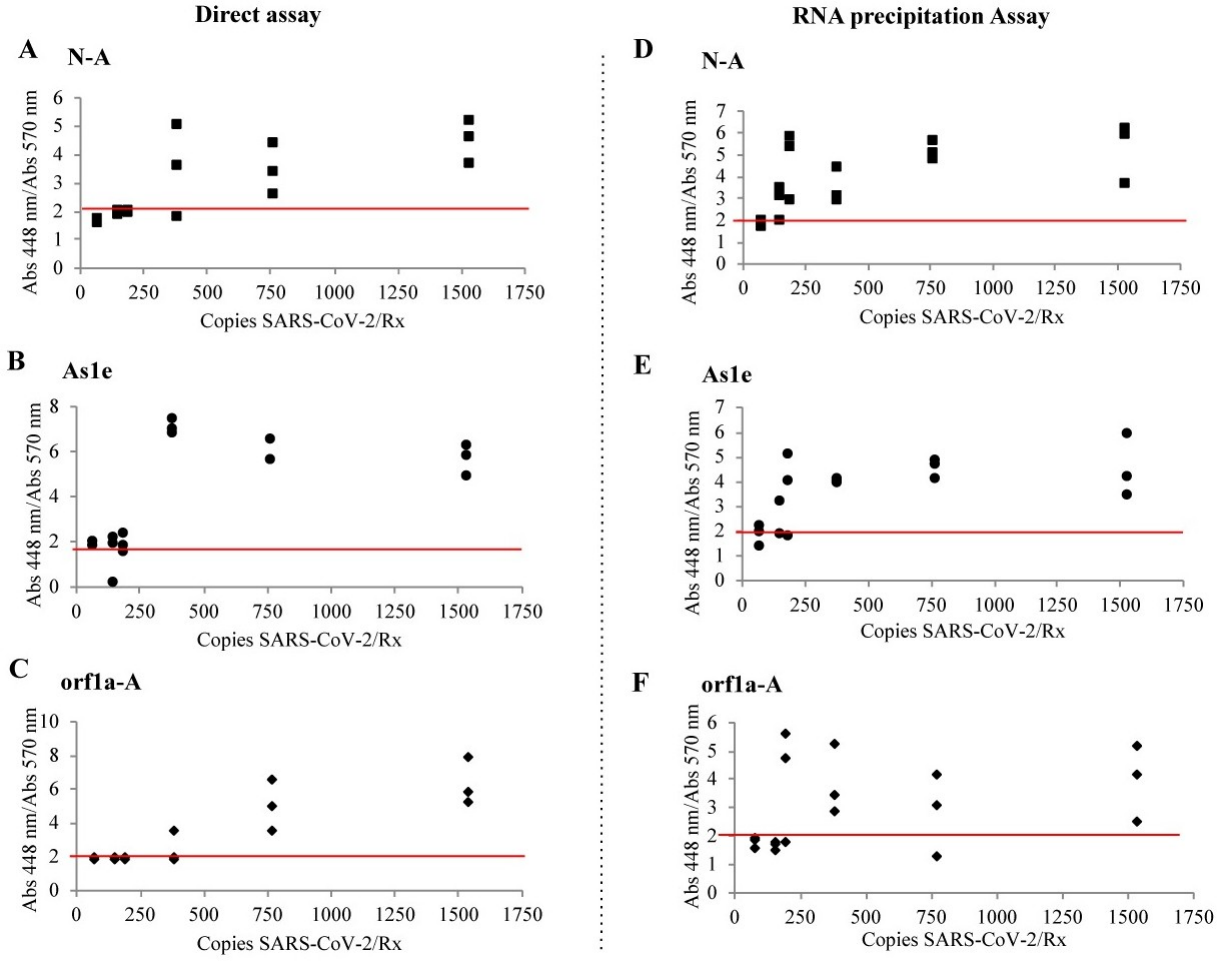
Limit of detection for SARS-CoV-2 spiked in VTM. VTM medium was spiked with different concentrations of SARS-CoV-2. Samples treated with Inactivation buffer and RNA secure (Direct assay: A, B, C) and RNA precipitated samples following the HMS Assay (RNA precipitation Assay: D,E,F) were added in the LAMP reaction with samples for (A,D) oligonucleotide NEB Gene N-A, (B,E) oligonucleotide HMS Assay 1e and (C,F) oligonucleotide NEB orf1a-A and incubated 30 minutes at 65°C. Each dot represents an individual experiment, n = 3. Critical value threshold was set at 2 (red line).

For the direct assay experiments, VTM was spiked with SARS-CoV-2 and inactivated with 100x inactivation buffer. The inactivation buffer has a final concentration of 0.05% SDS to help solubilize the virus membrane, rendering the virus non-infectious, as well as providing additional protection for RNA and DNA [25–27]. According to the direct assays protocol, we transferred 1 μL of the treated sample (Fig 3A–3C) to PCR tubes containing the RT-LAMP reaction media previously prepared with primers for NEB Gene N-A (A, D), HMS Assay 1e (B, E) and NEB orf1a-A (C, F) and incubated it 30 minutes at 65°C. In the direct assay (no RNA-precipitation), the limit of detection was 769 copies/μL (769 copies per reaction) with NEB Gene N-A and NEB orf1a-A primers, and 385 copies/mL (385 copies per reaction) when using the HMS Assay 1e primers (Fig 3A–3C, S2A Fig).

To determine if sensitivity was sacrificed by omitting RNA purification, we tested the RT-LAMP assay with a protocol where the viral RNA was isolated. We modified an inexpensive silica bead-based (“glassmilk”) method to isolate nucleic acids known as the HMS Assay [17, 28] (Fig 3D–3F, S2B Fig). 0.75 mL of VTM were spiked with diferent number of copies of SARS-CoV-2. We added the 100x inactivation solution supplemented with a 25x non-enzymatic RNase inhibitor (RNAsecure^™^) and proteinase K [18]. We added an additional incubation step at 55°C for 15 min, and then as in the direct assay, samples were incubated at 95°C for 5 min and centrifuged at 5,000 xg for 30s. Protein pellet in these samples was considerably smaller than the obtained in the Direct Assay. After NaI RNA precipitation, we resuspended the smaple in 9 μL of 1X inactivation buffer. Three μL of RNA precipitated ssample were added to previously set up RT-LAMP reactions containing primers for NEB Gene N-A (Fig 5A and 5D), HMS Assay 1e (Fig 5B and 5E) and Actin (Fig 5C and 5F). Samples were incubated 30 minutes at 65°C. Using this protocol, we found the limit of detection to be 192 copies/reaction when using the NEB Gene N-A primers. When using the HMS Assay 1e primers, the limit of detection was 385 copies/reaction. When using the NEB orf1a-A primers, we successfully detected 1538 and 385 copies/reaction, but one sample unexpectedly gave a negative reading at 769 copies/reaction. Thus, the addition of an RNA purification step that increases the testing time for about 40 min, but yielded a 2 to a 5-fold increase in sensitivity depending on the oligonucleotides used.

### Direct *versus* RNA-purified methods for detection of SARS-CoV-2 in nasopharyngeal patient samples

**Fig 4:**
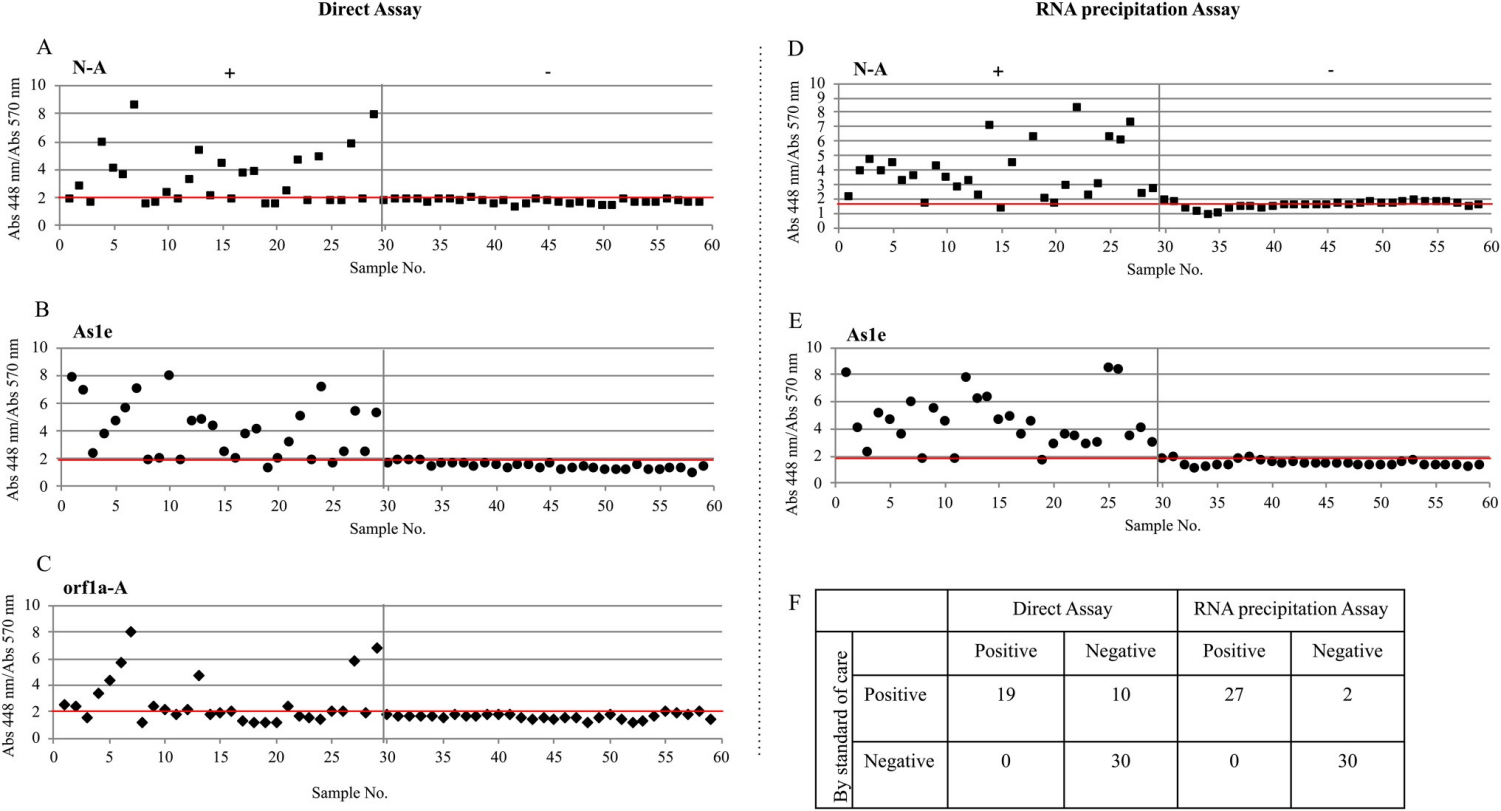
Detection of SARS-CoV-2 in clinical nasopharyngeal samples. NP patient samples in VTM were tested using the LAMP Direct Assay (A,B,C) or the RNA precipitation Assay following the HMS Assay (D,E) to detect SARS-CoV-2 using the (A,D) NEB Gene N-A, (B,E) HMS Assay 1e and the (C) NEB orf1a-A oligonucleotides. Positive and negative samples are paired with Table 1 (Direct Assay) and Table 2 (RNA precipitation Assay). (F) Two by two table showing positive and negative samples detected by the Direct Assay and the RNA precipitation Assay. Each dot represents an individual experiment. Critical value threshold was set at 2 (red line). Samples 1 to 29 are true positives by RT-qPCR. Samples from 30 to 59 are true negatives by RT-qPCR.

We tested both methods in a blind randomized assay of 29 positive and 30 negative patient-derived samples that were previously analyzed by RT-qPCR (Fig 4, Tables 1 and 2, S3 and S4 Figs).

**Table 1:** Comparison of RT-LAMP direct assay versus RT-qPCR obtained Ct values for 59 patient samples.

		LAMP					Ct		
No.	Name	N-A	Orf1a-A	As1e	Repeat	Result	N	Orf1	S
1 ^c^	5A2	-	+	+	+ (N)	+	20.197	21.933	23.296
2	6B7	+	+	+		+	10.361	13.661	13.423
3 ^a^	6B10	-	-	-		-	23.675	27.357	27.088
4	6B11	+	+	+		+	12.222	16.589	16.499
5	6B12	+	+	+		+	7.72	11.455	11.604
6	6F1	+	+	+		+	16.32	19.71	20.083
7	6C6	+	+	+		+	13.465	16.679	16.88
8 ^a^	6C1	-	-	-		-	26.384	29.887	29.957
9 ^b^	6C4	-	+	-		-	26.042	30.259	29.965
10	6E12	+	+	+		+	14.787	18.464	19.177
11 ^a^	6E9	-	-	-		-	23.298	26.505	25.839
12	6E7	+	+	+		+	14.727	18.749	18.453
13	6E2	+	+	+		+	14.678	18.152	17.864
14 ^c^	6E4	+	-	+		+	20.926	24.706	24.533
15 ^c^	9D2	+	-	+		+	23.877	25.261	26.907
16 ^a^	9D6	-	-	-		-	26.05	28.236	29.543
17 ^c^	9D7	+	-	+		+	21.82	23.246	24.531
18 ^c^	9D9	+	-	+		+	14.602	19.724	21.2
19 ^a^	9D10	-	-	-		-	24.184	25.817	26.844
20 ^b^	9D11	-	-	+	- (N)	-	23.463	25.039	27.681
21	9E7	+	+	+		+	17.659	19.745	20.783
22 ^c^	9E9	+	-	+		+	20.773	22.718	24.01
23 ^a^	9E10	-	-	-		-	27.542	30.628	31.202
24 ^c^	9E11	+	-	+		+	16.556	18.979	20.743
25 ^a^	9E12	-	-	-		-	29.672	32.071	35.659
26 ^b^	9C7	-	-	+	+ (N)	+	24.062	26.251	27.642
27	9C10	+	+	+		+	12.798	14.722	16.521
28 ^b^	8B10	-	-	+		-	25.986	26.214	27.702
29	8B12	+	+	+		+	17.605	16.74	19.045
30	46A4	-	-	-		-			
31	47E7	-	-	-		-			
32	47 E9	-	-	-		-			
33	47 G1	-	-	-		-			
34	46 B1	-	-	-		-			
35	46 B2	-	-	-		-			
36	47 H3	-	-	-		-			
37	47 H6	-	-	-		-			
38	47 H9	-	-	-	-	-			
39	47H12	-	-	-		-			
40	47 C6	-	-	-		-			
41	46 A5	-	-	-		-			
42	46 A 8	-	-	-		-			
43	46 A11	-	-	-		-			
44	46 D6	-	-	-		-			
45	47 A3	-	-	-		-			
46	47 B5	-	-	-		-			
47	46 B4	-	-	-		-			
48	46 B12	-	-	-		-			
49	47 E4	-	-	-		-			
50	47 G8	-	-	-		-			
51	47 G10	-	-	-		-			
52	46 C1	-	-	-		-			
53	46 C5	-	-	-		-			
54	46 C10	-	-	-		-			
55	46 C12	-	-	-	-	-			
56	46 C5	-	-	-		-			
57	47 A9	-	-	-		-			
59	46 A2	-	-	-		-			
59	46 A7	-	-	-		-			
Tot+						19/29			

59 de-identified patient samples remnant from the diagnostic swab samples were tested using the RT-LAMP Direct Assay. Samples 1–29 are positive and samples 30–59 are negative. The symbol in the simple letter indicates:

^a^ RT-qPCR positive samples that could not be detected by RT-LAMP;

^b^ RT-qPCR positive samples that were detected for 1 out of 3 oligonucleotide pairs by RT-LAMP; and

^c^ RT-qPCR positive samples that were detected for 2 out of 3 oligonucleotide pairs by RT-LAMP.

Sample numbers that do not have any additional symbol indicate RT-qPCR positive samples that were successfully detected by RT-LAMP. Ct values where obtained from RNA purificated samples using Qiagen QIAamp Viral or PerkinElmer chemagen Viral 300 kits, followed by RT-qPCR in an ABI QuantStudio 12K Flex instrument using the ThermoFisher TaqPath COVID-19 Combo Kit that detects genes N, Orf1 and S. RT-LAMP reactions are imaged in S3 Fig and absorbance measurements graphics are shown in Fig 4.

**Table 2:** Comparison of RT-LAMP precipitation assay versus RT-qPCR obtained Ct values for 59 patient samples.

		LAMP				Ct		
No.	Name	N-A	As1e	Repeat	Result	N	Orf1	S
1	5A2	+	+	+(N)	+	20.197	21.933	23.296
2	6B7	+	+		+	10.361	13.661	13.423
3	6B10	+	+	+(As1e)	+	23.675	27.357	27.088
4	6B11	+	+		+	12.222	16.589	16.499
5	6B12	+	+		+	7.72	11.455	11.604
6	6F1	+	+		+	16.32	19.71	20.083
7	6C6	+	+		+	13.465	16.679	16.88
8 ^a^	6C1	-	-		-	26.384	29.887	29.957
9	6C4	+	+		+	26.042	30.259	29.965
10	6E12	+	+		+	14.787	18.464	19.177
11 ^c^	6E9	+	-	+(As1e)	+	23.298	26.505	25.839
12	6E7	+	+		+	14.727	18.749	18.453
13	6E2	+	+		+	14.678	18.152	17.864
14	6E4	+	+		+	20.926	24.706	24.533
15 ^c^	9D2	-	+	+(N)	+	23.877	25.261	26.907
16	9D6	+	+		+	26.05	28.236	29.543
17	9D7	+	+		+	21.82	23.246	24.531
18	9D9	+	+		+	14.602	19.724	21.2
19 ^a^	9D10	-	-	-(N)	-	24.184	25.817	26.844
20 ^c^	9D11	-	+	+(N)	+	23.463	25.039	27.681
21	9E7	+	+		+	17.659	19.745	20.783
22	9E9	+	+	+(As1e)	+	20.773	22.718	24.01
23	9E10	+	+	+(N)	+	27.542	30.628	31.202
24	9E11	+	+		+	16.556	18.979	20.743
25	9E12	+	+		+	29.672	32.071	35.659
26	9C7	+	+		+	24.062	26.251	27.642
27	9C10	+	+		+	12.798	14.722	16.521
28	8B10	+	+		+	25.986	26.214	27.702
29	8B12	+	+		+	17.605	16.74	19.045
30	46A4	-	-		-			
31	47 E7	-	-		-			
32	47 E9	-	-		-			
33	47 G1	-	-		-			
34	46 B1	-	-		-			
35	46 B2	-	-		-			
36	47 H3	-	-		-			
37	47 H6	-	-		-			
38	47 H9	-	-		-			
39	47H12	-	-		-			
40	47 C6	-	-		-			
41	46 A5	-	-		-			
42	46 A 8	-	-		-			
43	46 A11	-	-		-			
44	46 D6	-	-		-			
45	47 A3	-	-		-			
46	47 B5	-	-		-			
47	46 B4	-	-		-			
48	46 B12	-	-		-			
49	47 E4	-	-		-			
50	47 G8	-	-		-			
51	47 G10	-	-		-			
52	46 C1	-	-		-			
53	46 C5	-	-		-			
54	46 C10	-	-		-			
55	46 C12	-	-		-			
56	46 C5	-	-		-			
57	47 A9	-	-		-			
58	46 A2	-	-		-			
59	46 A7	-	-		-			
Tot					27/29			

59 de-identified patient samples remnant from the diagnostic swab samples were tested using the RT-LAMP Direct Assay. Samples 1–29 are positive and samples 30–59 are negative. The symbol in the simple letter indicates:

^a^ RT-qPCR positive samples that could not be detected by RT-LAMP;

^b^ RT-qPCR positive samples that were detected for 1 out of 3 oligonucleotide pairs by RT-LAMP; and

^c^ RT-qPCR positive samples that were detected for 2 out of 3 oligonucleotide pairs by RT-LAMP. Sample numbers that do not have any additional symbol indicate RT-qPCR positive samples that were sucesfully detected by RT-LAMP. Ct values where obtained from RNA purificated samples using Qiagen QIAamp Viral or PerkinElmer chemagen Viral 300 kits, followed by RT-qPCR in an ABI QuantStudio 12K Flex instrument using the ThermoFisher TaqPath COVID-19 Combo Kit that detects genes N, Orf1 and S. RT-LAMP reactions are imaged in S4 Fig and absorbance measurements graphics are shown in Fig 4.

The Direct Assay (*i*.*e*., without RNA purification) successfully detected 17/29 positive samples when using the NEB Gene N-A oligonucleotides (Fig 4A), 21/29 positive samples when using the HMS Assay 1e oligonucleotides (Fig 4B), and 14/29 positive samples when using the NEB orf1a-A oligonucleotides (Fig 4C). The samples that scored positive for all 3 genes where those that had the lowest Ct values (≤20) determined by RT-qPCR, indicating a high number of viral RNA copies (Table 1, No symbol). The seven samples that were positive for 2 out of 3 genes, were considered positive and correlated with the mid-range Ct values (Table 1, c). The five samples that gave one out of 3 genes positive were repeated with the NEB Gene N-A or the HMS Assay 1e oligonucleotides and in only one case (27, 9C7) the sample showed positive detection (Table 1, b). The seven samples with the highest Ct’s (≥24) which corresponds to a lower viral load, gave negative results for all oligonucleotides (Table 1, a). Overall, the simple method had a 65.5% (95% confidence interval (45.7, 82.1)) sensitivity as compared to the standard clinical (*i*.*e*. with RNA purification) RT-qPCR test. No false positives were detected.

RNA precipitation prior to the RT-LAMP (Fig 4D and 4E, Table 2, S4 Fig) detected 25/29 positive samples for the NEB Gene N-A oligonucleotides and 26/29 positive samples for or the HMS Assay 1e oligonucleotides. We did not use NEB orf1a-A oligonucleotides due to the above-noted inconsistency in previous tests and the results obtained by others [17]. The samples that gave positive results with only one primer set were those with the highest Ct values, indicating a low number of viral RNA copies (Table 2, c). Samples with indeterminate results were repeated and amplification was confirmed (Fig 4D and 4E). No false positives were detected, and method sensitivity increased to 93.1% (95% confidence interval (77.2%, 99.2%)) indicating that concentrating the sample and precipitating RNA substantially enhances performance. However, this protocol required additional time and more hands-on manipulation for the sample precipitation, which should be considered versus the cost of an RNA precipitation kit.

### Comparison of direct *versus* RNA-purified methods for detection of SARS-CoV-2 in spiked saliva

Saliva-based tests do not require a certified swab, VTM, or a skilled worker to take samples. However, when using saliva, we found that the RT-LAMP test worked well for saliva samples with a neutral to basic pH (up to 7.0–7.4), but acidic saliva (less than pH 6.8) gave false positive results. To address this problem, we increased the pH of the inactivation buffer from 8.5 to 11. The increase in pH did not affect the RT-LAMP test results when using basic saliva, however, we noted that the ratios between the readings at 447/570 nm were consistently lower as compared to the original low pH buffer.

**Fig 5:**
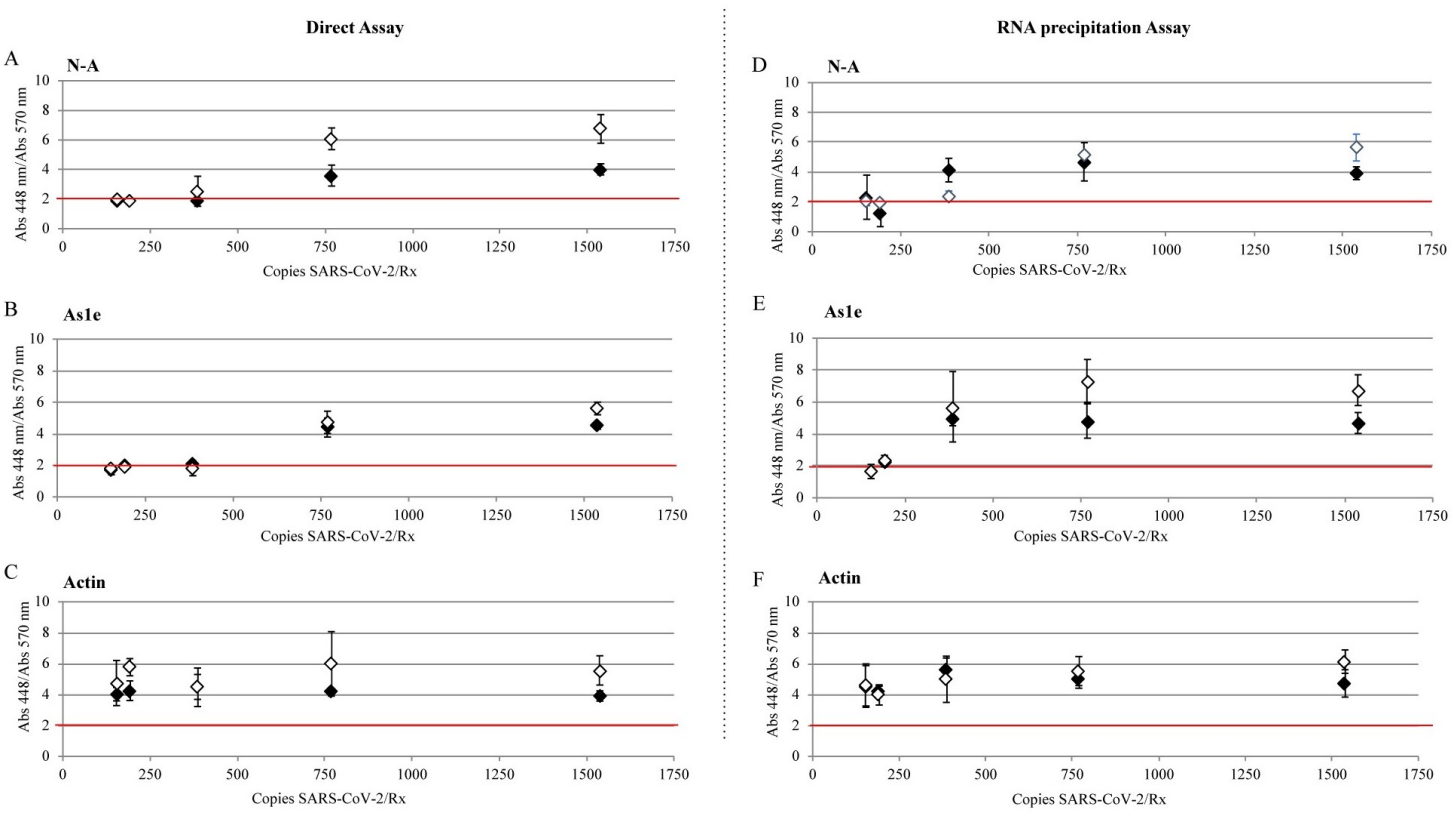
Limit of detection for SARS-CoV-2 spiked in saliva. Saliva samples were spiked with different concentrations of SARS-CoV-2. Samples treated with Inactivation buffer and RNA secure (Direct Assay: A, B, C) and RNA precipitated samples (RNA precipitation Assay: D,E,F) were added in the LAMP reaction with oligonucleotides (N-A: A,D) NEB Gene-N-A, (As1e: B,E) HMS Assay 1e, (C,F) and Actin and incubated 30 minutes at 65°C. Positive threshold was set at 2 (red line). ♦ saliva sample pH 7.4, ♢ saliva sample pH 6.7. Values represent the mean ± S.D. of n = 3 for each concentration.

We tested the direct (Fig 5A–5C, S5A Fig) and the RNA precipitation assays (Fig 5D–5F, S5B Fig) with known concentration of copies of SARS-CoV2 in saliva with the new high pH buffer and found the limit of detection for both, the NEB Gene N-A and the HMS Assay 1e oligonucleotides to be 769 copies/reaction for the direct method and 386 copies/reaction for the RNA precipitation Assay.

**Fig 6:**
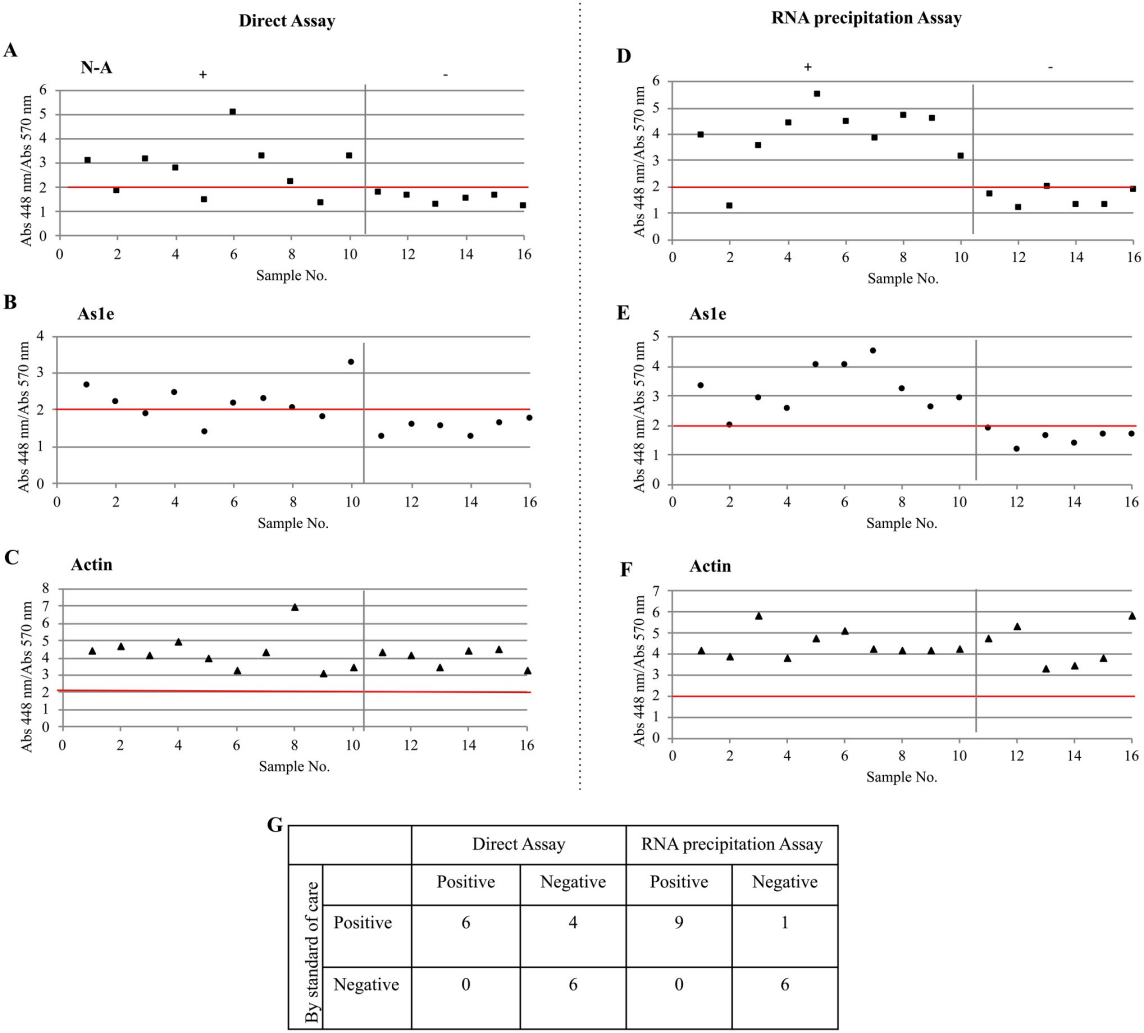
Detection of SARS-CoV-2 in clinical nasopharyngeal samples in virus transport medium diluted in saliva. Nasopharyngeal patient samples in VTM were diluted in saliva and tested using the LAMP Direct Assay (A,B,C) or the RNA precipitation Assay (D,E,F) to detect SARS-CoV-2. (G) Two by two table showing true positive and negative samples detected by the Direct Assay and the RNA precipitation Assay. Each dot represents an individual experiment. Critical value threshold was set at 2 (red line). Samples 1 to 10 are true positives by RT-qPCR. Samples from 11 to 16 are true negatives by RT-qPCR. Positive and negative samples are paired with Table 3.

We then assayed mock samples by diluting 50 μL of randomly chosen NP-positive and -negative patient-derived samples in both acidic and alkaline saliva samples (Fig 6, Table 3, S6 Fig). The testing group included 10 positive samples and 6 negative samples. The direct assay detected 7/10 positive samples when using both the NEB Gene N-A and the HMS Assay 1e oligonucleotides (Fig 6A and 6B). The three non-detected samples had the highest Ct values when measured by RT-qPCR (Table 3). The RNA precipitation assay detected 9/10 positive samples for both NEB Gene N-A and the HMS Assay 1e oligonucleotides (Fig 6D and 6B). Actin-based primers were used as a positive control (Fig 6C and 6F) [21]. We detected 90% of the samples but values were lower than in the samples that used VTM directly making the use of a spectrophotometer obligatory. Sensitivy of these methods in saliva was 60.0% (95% confidence interval (26.2%, 87.8%)) and 90.0% (95% confidence interval (55.5%, 99.8%)) respectively, while showing no false positives.

**Table 3:** Correlation between LAMP direct and precipitation methods and RT-qPCR tests for nasopharyngeal in VTM samples using saliva as transport medium.

		LAMP					Ct		
		Direct		Precipitation					
No.	Name	N	As1	N	As1	Result	N	Orf1	As1e
7	6C6	+	+	+	+	+	13.465	16.679	16.88
8 ^a^	6C1	-	+	-	-	-	26.384	29.887	29.957
11 ^c^	6E9	+	-	+	+	+	23.298	26.505	25.839
17	9D7	+	+	+	+	+	21.82	23.246	24.531
19 ^c^	9D10	-	-	+	+	-/+	24.184	25.817	26.844
20	9D11	+	+	+	+	-	23.463	25.039	27.681
21	9E7	+	+	+	+	+	17.659	19.745	20.783
22	9E9	+	+	+	+	+	20.773	22.718	24.01
27 ^c^	9C7	-	-	+	+	-/+	24.062	26.251	27.642
28	9C10	+	+	+	+	+	12.798	14.722	16.521
41	47 C6	-	-	-	-	-			
42	46 A5	-	-	-	-	-			
45	46 D6	-	-	-	-	-			
46	47 A3	-	-	-	-	-			
47	47 B5	-	-	-	-	-			
58	47 A9	-	-	-	-	-			

16 de-identified patient samples remnant from the diagnostic swab samples were mixed with saliva samples from healthy, consenting adult volunteers and tested for SARS-Co-V-2 using the RT-LAMP Direct Assay and the RNA precipitation methods. The symbol in the simple letter indicates:

^a^ RT-qPCR positive samples that could not be detected by RT-LAMP;

^b^ RT-qPCR positive samples that were detected for 1 out of 3 oligonucleotide pairs by RT-LAMP; and

^c^ RT-qPCR positive samples that were detected for 2 out of 3 oligonucleotide pairs by RT-LAMP. Sample numbers that do not have any additional symbol indicate RT-qPCR positive samples that were sucesfully detected by RT-LAMP. Ct values where obtained from RNA purificated samples using Qiagen QIAamp Viral or PerkinElmer chemagen Viral 300 kits, followed by RT-qPCR in an ABI QuantStudio 12K Flex instrument using the ThermoFisher TaqPath COVID-19 Combo Kit that detects genes N, Orf1 and S. RT-LAMP reactions are imaged in S6 Fig and absorbance measurements graphics are shown in Fig 6.

## Discussion

In an attempt to choose a robust and easy-to-perform test for SARS-CoV-2 for use in point-of-care settings, we tested several published RT-LAMP assay methods. We introduced modifications to simplify the procedure while maintaining high sensitivity and reliability. These modifications included changes in the pH of the RT-LAMP buffers, omission of an RNA purification step.

In accordance to Huang et al. [29], RT-LAMP was able to detect up to 2 copies of directly spiked SARS CoV-2 RNA per reaction showing it is as reliable as RT-qPCR and any variation on positive or negative results may come from sample handling and RNA isolation and stabilization methods. Using the Direct assay, we were able to detect the SARS CoV-2 RNA in 385 or 769 copies per reaction for the HMS Assay 1e and for the NEB Gene N-A, and the NEB orf1a-A oligonucleotides respectively. The precipitated method was more sensitive detecting between 192 and 385 copies per reaction for the NEB Gene N-A, and for the HMS Assay 1e and the NEB orf1a-A oligonucleotides respectively. These number of copies detected are comparable to those reported by others, and slightly lower than the sensitivity obtained using RNA column purification plus qRT-PCR that goes down to 10–15 copies per reaction (RT-qPCR Ct’s ≤ 37) [12, 17, 29] and is considerably more expensive. Consistent with other reports, direct spiked RT-LAMP test without RNA purification detects from 50 to 400 copies per reaction [17, 18, 30, 31] and Ct values below 24–26. Adding a column RNA purification step to ther RT-LAMP increases sensibility to 10–30 copies per reaction [13, 19, 32], which was lower than what we detected. The HMS Assay (glass milk precipitation method) reports 1–2 copies per μl of an initial 500 μL sample [17], by modifying the methos adding detergents and an RNA protective agent we were able to detect 0.6 to 1.54 copies per μl of an initial 250 μL sample which corresponds to samples with RT-qPCR Ct values of ~29.

The RT-LAMP assay has several potential advantages over qRT-PCR methods. First, RT-LAMP amplifies DNA fragments at a constant, modest temperature, obviating the need for a thermo-cycler. RT-LAMP also typically has higher DNA yields than common PCRs, since there is no bind, amplify, and release cycle [33]. Finally, during nucleic acid synthesis, the binding of each nucleotide to the DNA growing strand releases a proton, acidifying the medium [34] (Fig 1A). Therefore, product accumulation during RT-LAMP may be evaluated using common pH sensitive dyes such as phenol red, which changes from a pink/red tone at pH 8 to yellow as pH acidifies (Fig 1A) [34]. Other inexpensive pH sensitive dyes such as cresol red, neutral red and m-cresol purple have also been used to track DNA amplification [34].

Direct detection of SARS-CoV-2 in the absence of RNA purification was possible, but the sensitivity was reduced from 93% to 65% when compared to assays where RNA was first purified. In practical terms, this relative lack of sensitivity may be acceptable in certain circumstances, as the test is inexpensive and easy to perform it may be applied multiple times if required, and tends to give false negative only for low-titer samples that likely correlate with less transmissibility and/or less severity of disease [35–37]. The negative predictive value for the direct test method is 75.0% (95% confidence interval (58.8%, 87.3%)), which means that even if this test is negative, there is still a 25% chance of being sick, and it’s directly correlated with the virus load found in the patient. The negative predictive value for the precipitation method is 93.8% (95% confidence interval (79.2%, 99.2%)), which means that if the test is negative, there is still a 6% chance of being sick. For both methods specificity and positive predictive values were 100%.

The use of saliva samples in place of NP samples would represent a third potential improvement. Saliva is easy to obtain and we found that it can be kept at ambient temperature for periods up to 30 days [13, 15, 16]. This feature may circumvent the so-called refrigeration barrier, described for sub-Saharan regions, or any location that lacks adequate refrigerating facilities. We found that acidic saliva samples complicate testing as the assay readout is based on acidification as a result of DNA amplification and giving a high number of false positives. We successfully addressed this issue by simply increasing the initial pH of the inactivation buffer from 8.5 to 11. Even if it’s a dramatic modification in the initial pH, this buffer is diluted 100X in the saliva sample and further diluted to use in the RT-LAMP reaction. A higher pH helps maintain the neutral pH and color of the reaction buffer containing phenol red but still allows medium acidification and color change when there is a DNA amplification reaction. Other groups have avoided the pH issue by using fluorescent labels to follow DNA amplification which slightlty increases the cost and needs specialized equipment for detection [32, 38].

We were not able to get saliva samples from sick patients, but we tested the nasopharyngeal samples using saliva as a vehicle. The direct detection method sensitivity was 60% (95% confidence interval (26.2%, 87.8%), which is also considerably lower than the 93% (95% confidence interval (55.5%, 99.8%)) sensitivity of the assays where RNA was first purified. In practical terms, this relative lack of sensitivity may be acceptable in certain circumstances, as the test is inexpensive and easy to perform it may be applied multiple times if required, and tends to give false negative only for low-titer samples that likely correlate with less transmissibility and/or less severity of disease [35–37]. The negative predictive value for the direct test method is 60%, which means that even if this test is negative, there is still a 40% chance of SARS-CoV2 infection. The negative predictive value for the precipitation method is 90%, which means there would only be a 10% chance of being sick. For both methods specificity and positive predictive values were is 100%.

We envision a few additional modifications that might make the RT-LAMP-based self-testing procedure more suitable for point of care use. First, the enzymes (reverse transcriptase and *BstI* DNA polymerase), the buffer and the phenol red needed for the RT- LAMP reaction can be acquired in lyophilized form, allowing storage at room temperature [39, 40]. Second, small transfer loops could conceivably be used in place of micropipeting devices, allowing a reasonably accurate transfer of microliter volumes in settings that lack sophisticated equipment [41]. Third, pooled testing has been successfully demonstrated for RT-LAMP-based SARS-CoV-2 detection, further reducing costs while increasing output [40].

## Supporting information

S1 Fig LAMP assay limit of detection. Direct SARS-CoV-2 virus was diluted in inactivation buffer and LAMP using oligonucleotide pairs NEB Gene N-A (N-A), HMS Assay 1e (As1e) and NEB orf1a-A (orf1a-A). Controls using no virus were also monitored. LAMP tests were incubated in PCR tubes at 65°C for 30 minutes in a Bio-Rad thermocycler and the resulting reaction was imaged.
(PDF)

S2 Fig Limit of detection for SARS-CoV-2 spiked VTM. VTM medium was spiked with different concentrations of SARS-CoV-2. 1 μL of samples treated with A) the Direct Assay: Inactivation buffer and RNA secure and the B) RNA precipitation Assay were added in the LAMP reaction with oligonucleotides NEB Gene N-A (N-A), HMS Assay 1e (As1e) and NEB orf1a-A (orf1a-A) and incubated 30 minutes at 65°C and the resulting reaction was imaged.
(PDF)

S3 Fig Direct assay of SARS-CoV-2 in clinical nasopharyngeal samples in VTM. NP patient samples in VTM were tested using the LAMP direct assay to detect SARS-CoV-2 with oligonucleotides NEB Gene N-A (N-A), HMS Assay 1e (As1e) and NEB orf1a-A (orf1a-A) and incubated 30 minutes at 65°C and the resulting reaction was imaged. Positive and negative samples are paired with Table 1.
(PDF)

S4 Fig Detection of SARS-CoV-2 in clinical NP samples in VTM precipitating using the RNA precipitation assay. NP patient samples in VTM were precipitated with the HMS modified method and then tested with LAMP to detect SARS-CoV-2 with the NEB Gene N-A (N-A) and HMS Assay 1e (As1e) oligonucleotides. Samples were incubated 30 minutes at 65°C and the resulting reaction was imaged. Positive and negative samples are paired with Table 2.
(PDF)

S5 Fig SARS-CoV-2 limit of detection in saliva samples using the LAMP assay. Two saliva samples, of pH 6.7 and 7.4 were was spiked with different concentrations of SARS-CoV-2. Samples treated with A) the Direct Assay B) the RNA precipitation assay were tested with the NEB Gene N-A (N-A) and HMS Assay 1e (As1e) and Actin oligonucleotides. The resulting reaction was imaged after a 30 minute incubation at 65°C.
(PDF)

S6 Fig Detection of SARS-CoV-2 in clinical NP samples in VTM diluted in saliva. NP patient samples in VTM were diluted in saliva in a 1:5 ratio and tested using the (A) Direct Assay and the (B) RNA precipitation assay. LAMP test were done for tested with the NEB Gene N-A (N-A) and HMS Assay 1e (As1e) and Actin oligonucleotides. Positive and negative samples are paired with Table 3.
(PDF)
