# Supplementary figures and images for: Low saliva pH can yield false positives results in simple RT-LAMP-based SARS-CoV-2 diagnostic tests
Source: PLoS One. 2021 May 5;16(5):e0250202. doi: 10.1371/journal.pone.0250202 (PMC8099103; doi:10.1371/journal.pone.0250202)

SUPPLEMENTARY FIGURES

Copies/reaction

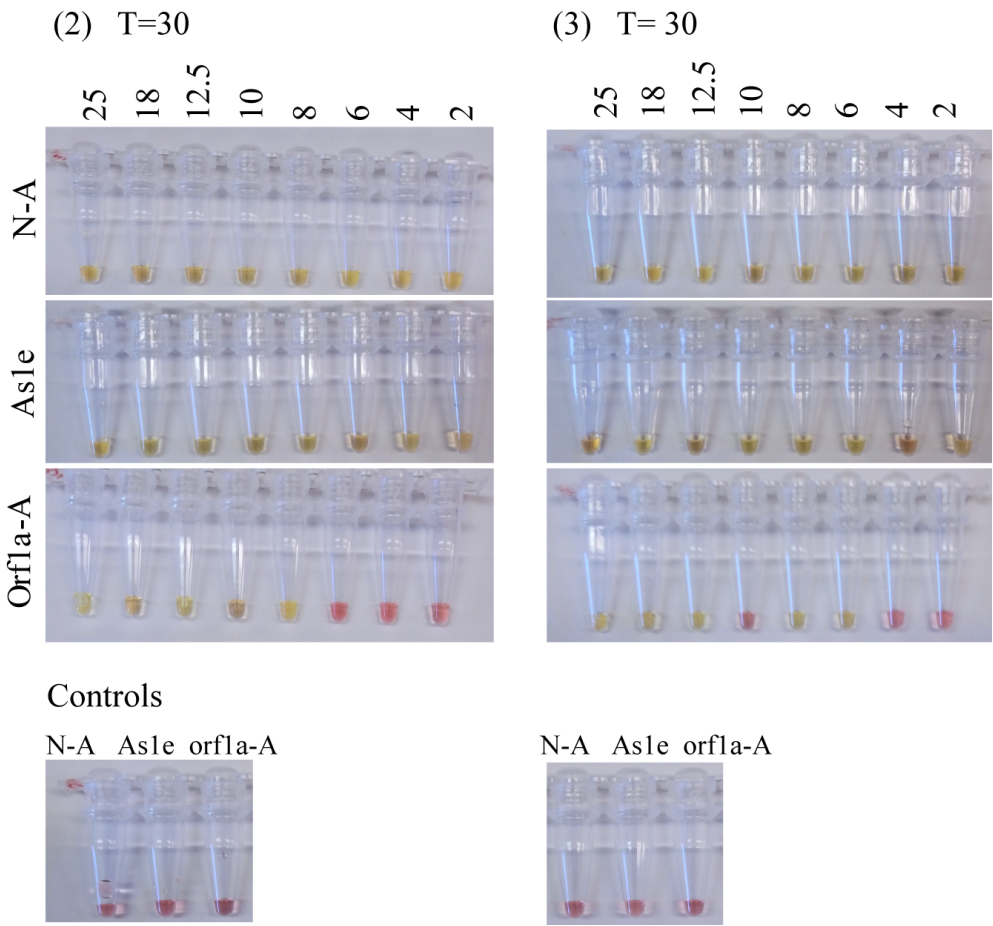

Supplement: S1 Fig — Direct SARS-CoV-2 virus was diluted in inactivation buffer and LAMP using oligonucleotide pairs NEB Gene N-A (N-A), HMS Assay 1e (As1e) and NEB orf1a-A (orf1a-A). Controls using no virus were also monitored. LAMP tests were incubated in PCR tubes at 65°C for 30 minutes in a Bio-Rad thermocycler and the resulting reaction was imaged. (PDF) [file pone.0250202.s001.pdf]

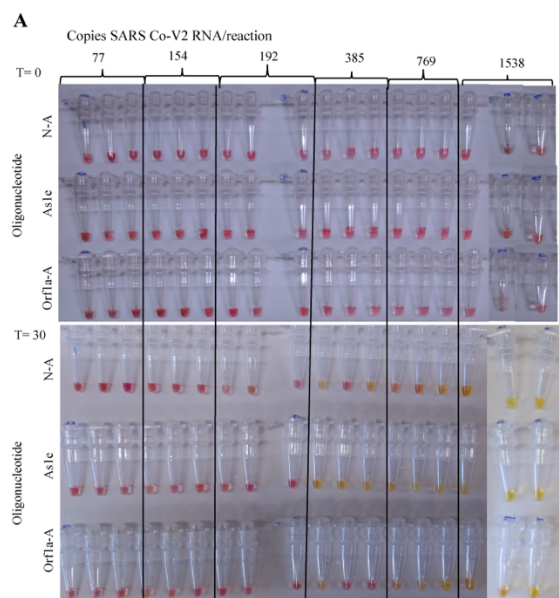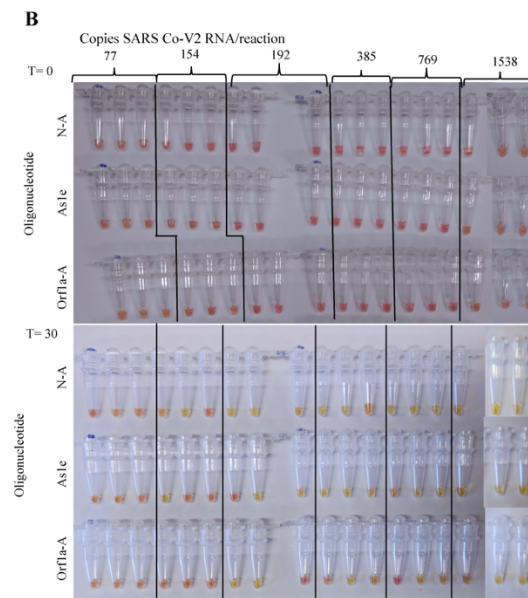

Supplement: S2 Fig — VTM medium was spiked with different concentrations of SARS-CoV-2. 1 μL of samples treated with A) the Direct Assay: Inactivation buffer and RNA secure and the B) RNA precipitation Assay were added in the LAMP reaction with oligonucleotides NEB Gene N-A (N-A), HMS Assay 1e (As1e) and NEB orf1a-A (orf1a-A) and incubated 30 minutes at 65°C and the resulting reaction was imaged. (PDF) [file pone.0250202.s002.pdf]

**A**

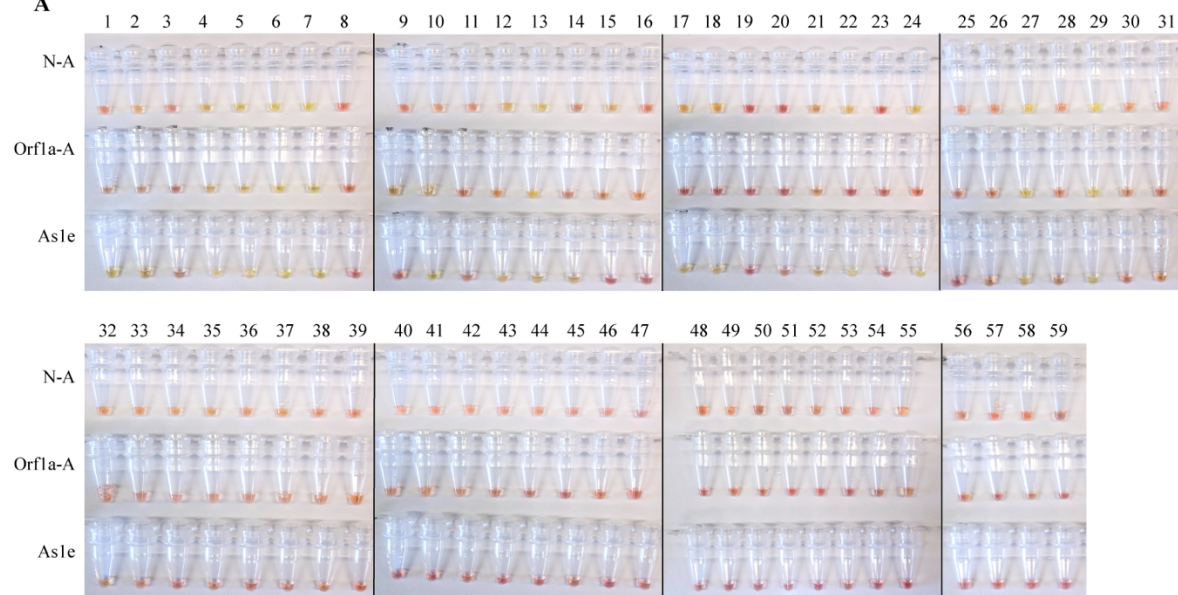

Supplement: S3 Fig — NP patient samples in VTM were tested using the LAMP direct assay to detect SARS-CoV-2 with oligonucleotides NEB Gene N-A (N-A), HMS Assay 1e (As1e) and NEB orf1a-A (orf1a-A) and incubated 30 minutes at 65°C and the resulting reaction was imaged. Positive and negative samples are paired with Table 1. (PDF) [file pone.0250202.s003.pdf]

**A**

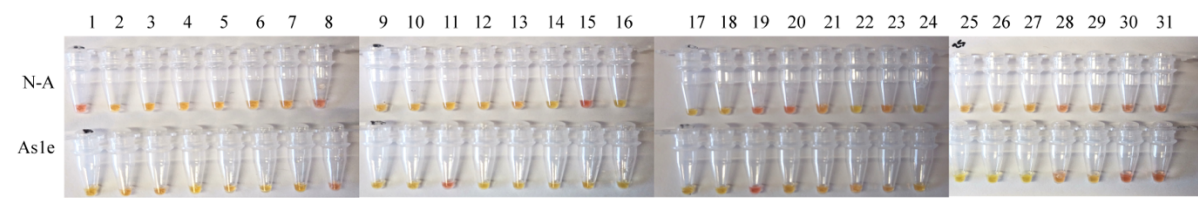

**B**

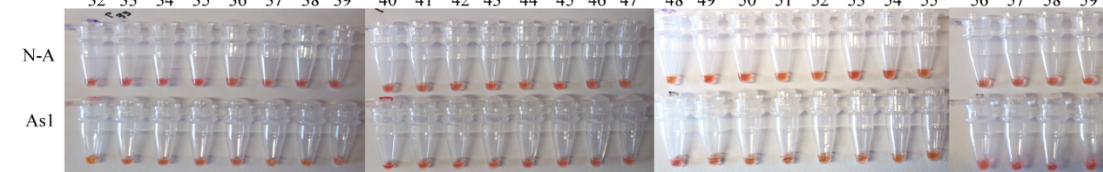

**C**

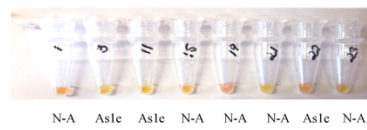

Supplement: S4 Fig — NP patient samples in VTM were precipitated with the HMS modified method and then tested with LAMP to detect SARS-CoV-2 with the NEB Gene N-A (N-A) and HMS Assay 1e (As1e) oligonucleotides. Samples were incubated 30 minutes at 65°C and the resulting reaction was imaged. Positive and negative samples are paired with Table 2. (PDF) [file pone.0250202.s004.pdf]

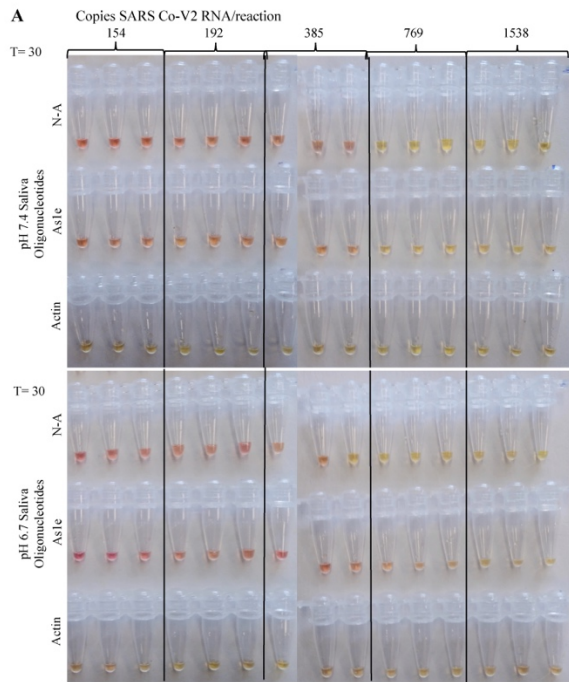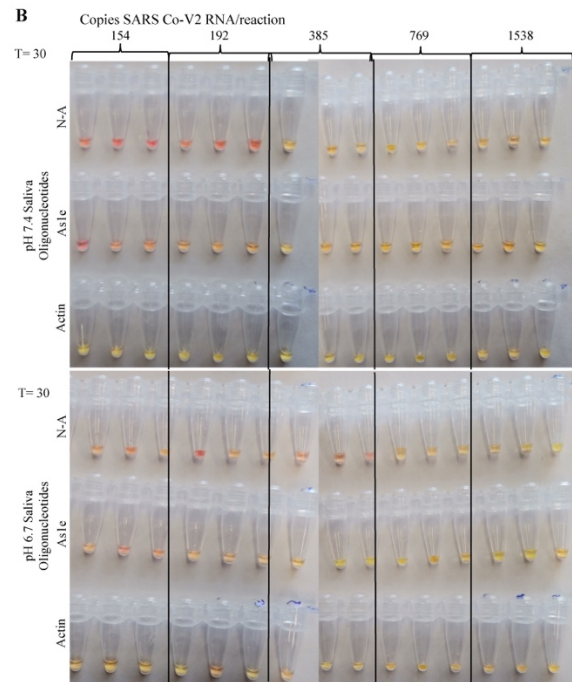

Supplement: S5 Fig — Two saliva samples, of pH 6.7 and 7.4 were was spiked with different concentrations of SARS-CoV-2. Samples treated with A) the Direct Assay B) the RNA precipitation assay were tested with the NEB Gene N-A (N-A) and HMS Assay 1e (As1e) and Actin oligonucleotides. The resulting reaction was imaged after a 30 minute incubation at 65°C. (PDF) [file pone.0250202.s005.pdf]

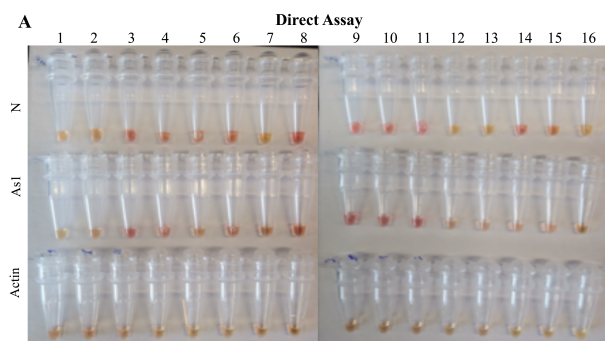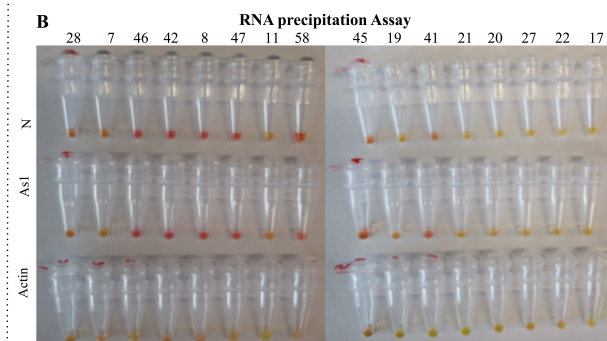

Supplement: S6 Fig — NP patient samples in VTM were diluted in saliva in a 1:5 ratio and tested using the (A) Direct Assay and the (B) RNA precipitation assay. LAMP test were done for tested with the NEB Gene N-A (N-A) and HMS Assay 1e (As1e) and Actin oligonucleotides. Positive and negative samples are paired with Table 3. (PDF) [file pone.0250202.s006.pdf]
